# Enhanced Detection of Atrial Fibrillation and Tachyarrhythmia Differentiation Using Atrial Electrograms in Single-chamber Implantable Cardioverter-defibrillators: A Retrospective Analysis

**DOI:** 10.19102/icrm.2025.16122

**Published:** 2025-12-15

**Authors:** Nithin Kumar Konanur Srinivasa, Canan Dilay Dirican, Atul Prakash

**Affiliations:** 1 Department of Cardiology, Saint Mary’s General Hospital, Passaic, NJ, USA

**Keywords:** Atrial electrograms, atrial fibrillation, single-chamber ICD, subclinical atrial fibrillation

## Abstract

Atrial fibrillation (AF) is a common comorbidity among patients with implantable cardioverter-defibrillators (ICDs). The presence of AF in this patient population is associated with increased mortality and morbidity. Dual-chamber ICDs allow for atrial arrhythmia detection; however, they have higher complication rates of implantation compared to single-chamber ICDs. Recent advances have enabled single-chamber ICDs to incorporate atrial electrogram (AEGM) sensing via a floating atrial dipole, potentially improving AF detection and arrhythmia discrimination. This study evaluates the effectiveness of AEGM-enabled single-chamber ICDs in detecting AF and differentiating tachyarrhythmias compared to single-chamber ICDs without AEGM and subcutaneous ICDs (S-ICDs). A retrospective analysis was done on 128 patients who received single-chamber ICDs between 2015 and 2024. Patients were stratified into three groups: (1) ICDs with AEGM, (2) ICDs without AEGM, and (3) S-ICDs. Baseline characteristics, comorbidities, arrhythmia events, and device data were collected. Numbers of ventricular tachycardia (VT), supraventricular tachycardia (SVT), and AF events were evaluated in all three devices. Outcomes measured included the detection of new and prior AF, tachycardia classification, initiation of anticoagulation, anticoagulants, AF ablation, congestive heart failure exacerbations, hospital admissions, and mortality. Statistical comparisons were made using logistic regression analysis. ICDs with AEGM (group 1, n = 69) detected AF in 42% (n = 29) of patients, including 13% (n = 9) with newly diagnosed, asymptomatic AF. In contrast, ICDs without AEGM (group 2, n = 34) detected AF only in patients with a prior history (n = 11), with no new cases identified. S-ICDs (group 3, n = 25) detected one new AF case, confirmed by external monitoring. The detection rate of new-onset subclinical AF was significantly higher in group 1 compared to groups 2 and 3 (*P* < .05). Among nine new AF patients in group 1, anticoagulation was started in four patients, anti-arrhythmics were initiated in four patients, two patients underwent AF ablation, and one patient underwent direct current cardioversion. VT events in all groups were analyzed, and the appropriateness of the therapy was confirmed in group 1 with the availability of AEGM and in groups 2 and 3 with EGM morphology and irregularity of the R–R intervals. There was no statistical difference among the groups with VT management (*P* > .05). In conclusion, single-chamber ICDs equipped with AEGM capabilities significantly enhance the detection of asymptomatic and new-onset AF. This has important implications for the management and prevention of AF-related complications in high-risk populations. Though there was no difference in VT defibrillation among all three groups, the validation of VT/SVT/AF was much more decisive in ICDs with AEGM.

## Introduction

Atrial fibrillation (AF) is the most common sustained cardiac arrhythmia in clinical practice and is associated with a five-fold increased risk of stroke, along with increased morbidity, mortality, and health care use.^1^ Implantable cardioverter-defibrillators (ICDs) are placed for the primary or secondary prevention of sudden cardiac death caused by ventricular tachyarrhythmias.^2^ Due to overlapping risk factors such as structural heart disease, coronary artery disease (CAD), hypertension, diabetes, and heart failure (HF), the prevalence of AF is particularly high in the population of patients indicated for ICD implantation.^3^

Standard single-chamber ICDs are designed primarily for ventricular arrhythmia detection and treatment and have one lead in the right ventricle. Dual-chamber ICDs have an additional atrial lead, which allows the detection of atrial arrhythmias; however, studies have shown that the implantation of dual-chamber ICDs is associated with higher rates of complications and in-hospital mortality.^4^

Recent technological advancements have enabled the development of single-chamber ICDs with integrated atrial sensing using a floating atrial dipole. These systems record atrial electrograms (AEGMs), which provide information about atrial activity and enable an accurate diagnosis of previously undetected AF and better discrimination of supraventricular and ventricular tachyarrhythmias in patients with ICDs.^5^ A significant proportion of AF episodes are asymptomatic, which makes the detection of the disease difficult and delays appropriate therapy such as anticoagulation or rhythm-control strategies.^6,7^ AF episodes that are identified by ICDs but not detected by standard electrocardiogram (ECG) monitoring are referred to as subclinical AF and are associated with increased mortality and risk of stroke.^7,8^

AEGMs enable continuous monitoring for AF and detection of subclinical AF episodes and improve distinguishing supraventricular tachycardias (SVTs) and ventricular tachycardias (VTs).^4,9^ By differentiating SVTs from VTs, AEGMs may reduce inappropriate ICD shocks, as demonstrated in the OPTION trial, to the OPTION trial, which have been linked to increased mortality, patient anxiety, and reduced device longevity.^10,11^ The reduction of inappropriate shocks with available AEGMs was, however, not significantly different in another analysis by Friedman et al.^12^

Although AEGMs offer several advantages, they are not included in all ICD platforms, and the impact of their inclusion—especially regarding AF detection—has not been fully established. This study aims to assess the real-world benefits of AEGMs in single-chamber ICDs by comparing AF detection rates and tachycardia discrimination in patients with ICDs who have AEGM capability versus those who do not, as well as patients with subcutaneous ICDs (S-ICDs). We hypothesize that devices with AEGM capability will be able to better detect new-onset AF and confirm the diagnosis in patients with pre-existing AF. Furthermore, the availability of recording AEGMs may reduce inappropriate shocks and help in correctly adjudicating a tachycardia event.

## Methods

We performed a retrospective chart review of 128 patients implanted with single-chamber ICDs between 2015 and 2024. All patients in our database with single-chamber ICDs were included in this analysis with no exceptions. The selection of the ICD and device programming protocol was dependent on the implanting physician. The selection of the device with respect to a device with AEGMs and a device without AEGMs was random and without bearing on the clinical characteristics of the patient. Patients with dual-chamber ICDs and biventricular ICDs were excluded. However, patients with documented sinus bradycardia and atrioventricular (AV) block did not receive a single-chamber ICD or S-ICD. S-ICDs were preferred for younger patients and those with a complicated vein architecture. Patients were stratified into three groups/cohorts based on the device type and AEGM capability. Baseline characteristics, comorbidities, arrhythmia events, and device data were collected.

**Figure 1:**
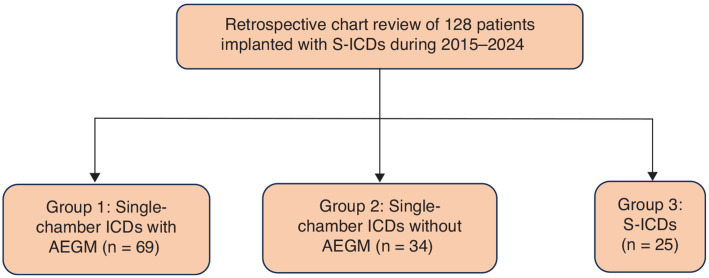
Flowchart showing stratification of patients into three cohorts based on the implantable cardioverter-defibrillator type and atrial electrogram capability for the retrospective analysis. *Abbreviations:* AEGM, atrial electrogram; ICD, implantable cardioverter-defibrillator; S-ICD, subcutaneous implantable cardioverter-defibrillator.

**Figure 2:**
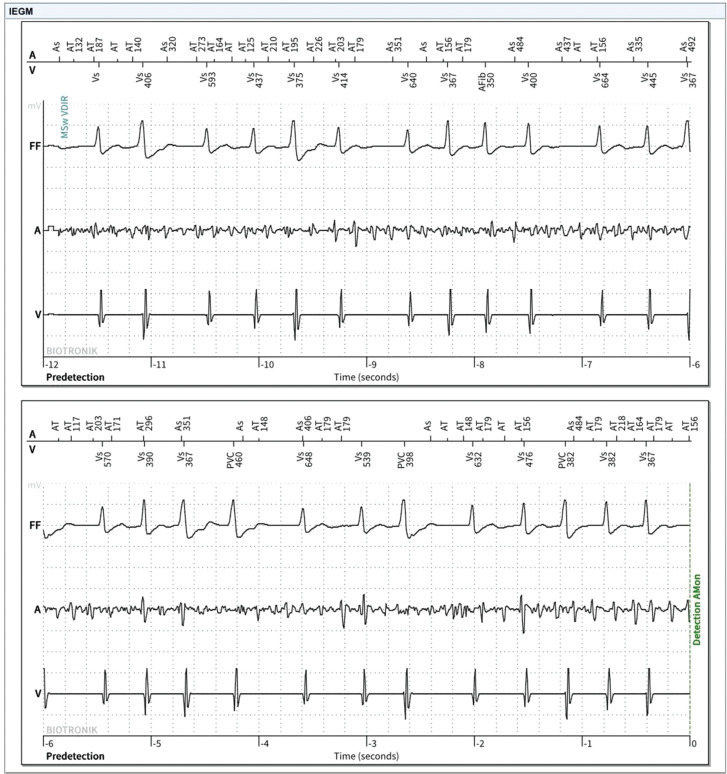
Intracardiac electrogram of one of the patients from cohort 1 diagnosed with new-onset atrial fibrillation. It highlights the irregular R–R interval and atrial electrogram with the atrial fibrillation picture.

We retrospectively analyzed the detection of AF by EGM validation and tachycardia events in patients with single-chamber ICDs with and without available AEGMs. Group 1 consisted of ICDs with AEGM, group 2 consisted of ICDs without AEGM, and group 3 consisted of S-ICDs **(Figure 1)**. AEGM with R–R variability was used to diagnose AF in cohort 1 **(Figure 2)**. In cohorts 2 and 3, event monitoring was performed if the patient had symptoms indicative of arrhythmias. SVT, VT, and ventricular fibrillation (VF) episodes were identified from device interrogation data.

Outcomes measured included AF detection (new vs. prior), tachycardia classification, and incidence of inappropriate therapy. Statistical comparisons between groups were made using regression analysis, with significance set at *P* < .05.

Institutional review board approval was obtained from the concerned governing body.

## Results

Group 1 included 69 patients with AEGM-enabled single-chamber ICDs and comorbidities that included hypertension, diabetes, hyperlipidemia, and CAD **(Table 1)**. Upon review, AF events were detected in 29 patients (42%) during follow-up; of these, 20 (29%) had a prior history of AF, while nine (13%) were newly identified. Eight patients were later diagnosed with clinical AF during follow-up. SVT occurred in six patients (9%), VT was found in 24 (34.7%), and VF was seen in 18 (26.5%). No inappropriate shocks were delivered, attributable to accurate tachycardia discrimination using AEGM data. The mean follow-up was 38 ± 10 months.

**Table 1:** Baseline Characteristics and Arrhythmia Events Among Patients Across the Three Device Cohorts

	ICDs with AEGM (n = 69)	ICDs Without AEGM (n = 34)	Subcutaneous ICDs (n = 25)
Age (years)	68 (38–86)	68 (40–96)	59.5 (25–81)
Female sex (%)	15 (22%)	9 (26%)	3 (12%)
Follow-up period (years)	3.12	2.67	2.76
History of hypertension	48 (70%)	25 (73%)	15 (60%)
History of dyslipidemia	27 (40%)	16 (47%)	9 (36%)
History of diabetes	14 (20%)	16 (47%)	6 (24%)
History of coronary artery disease	36 (52%)	15 (44%)	9 (36%)
Total patients with AF events on ICD	29 (42%)	11 (32%)	6 (24%)
New-onset AF patients detected by ICD	9 (13%)	0	1 (4%)
New-onset AF detected by any clinical means	8 (11.5%)	0	1 (4%)
SVT events	6 (9%)	4 (12%)	0
VT events	24 (34.7%)	15 (44%)	2 (8%)

*Abbreviations:* AEGM, atrial electrogram; AF, atrial fibrillation; ICD, implantable cardioverter-defibrillator; SVT, supraventricular tachycardia; VT, ventricular tachycardia.

Group 2 consisted of 34 patients without AEGM monitoring with various comorbidities **(Table 1)**. A retrospective review showed AF events in 11 (32%) patients, all of whom had a known history of AF prior to ICD implantation. No new-onset AF cases were detected during the follow-up period both clinically and during device evaluation. External event monitors and ECGs were employed for arrhythmia evaluation based on clinical suspicion.

Group 3 included 25 patients with S-ICDs with different comorbidities **(Table 1)**. Only one case of newly detected AF was observed, confirmed by external monitoring and clinically. Five other patients with AF events had a previous history. Appropriate therapy was delivered among the patients with VT and VF events in all three groups.

A comparative analysis of baseline characteristics of arrhythmia outcomes was conducted across all three cohorts. There was a statistically significant difference in age among cohort 3 (*P* < .05), with these patients being relatively younger than those in the other groups. The prevalence of diabetes was also significant (*P* < .05) across the groups, with the highest seen in cohort 2.

The detection of new-onset AF among all three cohorts was analyzed using the Firth penalized logistic regression, which provides stable estimates in small and rare event settings. The results showed a statistically significant lower probability of AF detection among cohort 2 (with non-AEGM devices) compared to cohort 1 (with AEGM devices) (*P* < .05). A comparison of cohort 3 (subcutaneous devices) and cohort 1 (AEGM devices) showed a reduced odds of association among cohort 3, but the difference was not statistically significant (*P* = .083). In contrast, there was no statistically significant difference in the prevalence of AF or SVT between cohorts.

The outcomes among patients with newly diagnosed AF detected by AEGM-enabled devices were evaluated in comparison with patients with a prior history of AF and AEGM-enabled ICDs. Anticoagulant initiation, anti-arrhythmic usage, AF ablation, direct current cardioversion, overall hospitalizations, HF-related hospitalizations, and mortality were evaluated. A comparative analysis of this is presented in **Table 2**.

**Table 2:** Comparison of Outcomes Among Patients with Device-detected New-onset Versus Prior History of Atrial Fibrillation in Cohort 1

Outcomes	Statistical Significance	*P* Value
Anticoagulation initiation	Significant	.027
Anti-arrhythmic initiation	Non-significant	.681
AF ablation	Non-significant	1.00
Direct current cardioversion	Non-significant	1.00
Overall hospitalization	Non-significant	.697
Heart failure exacerbation hospitalizations	Significant	.011
Death	Non-significant	1.00

*Abbreviations:* AEGM, atrial electrogram; AF, atrial fibrillation; ICD, implantable cardioverter-defibrillator.

Anticoagulation initiation was significantly more common among new-onset AF patients (*P* < .05), suggesting a more aggressive initiation of therapy. New-onset patients also experienced lower rates of congestive HF exacerbations requiring hospitalization (*P* < .05). No statistically significant differences were observed between both groups in the use of anti-arrhythmics, catheter AF ablation, direct current cardioversion, all-cause hospitalizations, and death.

## Discussion

In this retrospective study, we observed that single-chamber ICDs with AEGM capabilities were associated with a higher detection rate of AF, including asymptomatic and previous undiagnosed episodes. Among patients with no previous history of AF, 13% (n = 9) of the patients in group 1 were diagnosed with subclinical AF during follow-up. Eight patients among them were diagnosed with clinical AF following the development of symptoms. This underscores that continuous atrial monitoring with AEGM may improve the recognition of subclinical AF in patients with single-chamber ICDs. This result aligns with those of prior investigations, such as the Sensing Atrial High-Rate Episodes with Implantable Cardioverter-defibrillators (SENSE) study, which demonstrated that the detection of atrial high-rate episodes such as AF was significantly greater with single-chamber ICDs with a floating atrial dipole.^5^ Our analysis further substantiates the detection of asymptomatic previously undocumented AF by devices with AEGM recording capabilities.

The evaluation of outcomes among new and prior AF patients in group 1 highlighted significant use of anticoagulants. However, with anti-arrhythmics, the difference was not statistically significant. This decision may reflect increased clinician confidence with the diagnosis enabled by AEGMs. However, these decisions are also subject to clinical judgment, and causal inference cannot be drawn.

In patients with ICDs without AEGMs, the detection of AF relies on intermittent ECGs or patient-reported symptoms, which may result in underdiagnosis.^13^ This aligns with prior studies supporting a higher prevalence of subclinical AF among patients with cardiac implantable devices.^14^ Nonetheless, detection rates may be influenced by patient comorbidities.^15^ Considering that AF is associated with outcomes like thromboembolism and decompensation of HF, an early accurate diagnosis of AF and management may help with prognosis. The current study supports this, with reduced HF exacerbations requiring hospitalizations among new-onset AF patients with AEGM monitoring.

Currently, the evidence is not clear regarding the effectiveness of dual-chamber discrimination algorithms versus single-chamber discriminators in preventing inappropriate shocks. Several studies have demonstrated a decreased rate of inappropriate shocks in systems with AEGM compared to single-chamber ICDs, while Friedman et al. highlighted no significant difference.^12,16,17^ However, our study did not identify a significant difference in VT therapy appropriateness between groups. The ability to validate the tachycardia origin with atrial tracings may still offer clinical value in specific cases.

Some single-chamber ICDs without AEGM capabilities do have algorithms for atrial arrhythmia detection, but an accurate diagnosis solely relies on external monitors and occasional ECGs. SVT differentiation with just ventricular parameters increases the potential for misclassification, particularly in cases of rapid atrial arrhythmias such as atrial flutter or AV nodal re-entrant tachycardia.^18^ This reinforces the value of atrial data in improving device algorithms for arrhythmia classification.

The performance of S-ICDs (group 3) in our study reinforces the limitations of systems without intracardiac sensing capabilities. One patient in this cohort was diagnosed with AF following external monitoring. While these devices offer advantages in select populations, our findings highlight the potential diagnostic limitations in AF detection.

From a clinical perspective, the findings support the notion that AEGM-enabled single-chamber ICDs may offer incremental diagnostic benefit, especially for patients at high risk of AF without pacing indications. These AEGM ICDs provide a cost-effective and less-invasive alternative to dual-chamber systems while preserving diagnostic benefits. However, this must be balanced with device availability, operator comfort, cost, and patient selection. Future prospective studies are needed to validate whether enhanced AF detections with AEGM translate to meaningful improvements in outcomes such as stroke prevention, HF decompensation, or mortality. There is a need for a prospective randomized study with single-chamber ICDs to look at AF detection and discrimination of different tachyarrhythmias.

### Limitations

The study has its limitations, which need to be acknowledged. The retrospective design could lead to potential selection bias and patient interference. Operator-dependent device selection and programming may influence patient allocation and outcomes. Additionally, the sample size of the cohorts was relatively small, especially considering patients with S-ICDs, leading to difficulty in statistical subgroup analysis and detection of uncommon events. The selection of the devices with and without AEGM recording capabilities was not randomized but at the discretion of the implanting physician.

Second, the follow-up duration difference among groups may influence arrhythmia detection. Cohorts without AEGM monitoring might have had asymptomatic AF episodes, which may go undetected owing to the dependence of symptom-driven external monitoring.

Finally, the clinical decisions, including anticoagulant initiation and cardioversions in AF, were dependent on patient characteristics and physician judgment. Uniform implementation across all three cohorts is difficult and needs to be interpreted with caution. Long-term studies with uniform follow-ups and increased sample size are imperative before determining whether enhanced AF detection would lead to long-term improved prognosis.

## Conclusion

AEGM-enabled single-chamber ICDs help with the detection of asymptomatic and new-onset AF and allow for accurate arrhythmia discrimination. This has important implications for long-term patient management and prevention of AF-related complications. Increased use of AEGM-associated ICDs may enhance the diagnostic and therapeutic value of single-chamber ICDs.
